# Similar efficacy of broad-range ITS PCR and conventional fungal culture for diagnosing fungal infections in non-immunocompromised patients

**DOI:** 10.1186/s12866-016-0752-1

**Published:** 2016-06-28

**Authors:** Silvana K. Rampini, Andrea Zbinden, Roberto F. Speck, Guido V. Bloemberg

**Affiliations:** Klinik und Poliklinik für Innere Medizin, UniversitätsSpital Zürich, Universität Zürich, Rämistrasse 100, CH-8091 Zürich, Switzerland; Institut für Medizinische Mikrobiologie, Universität Zürich, Gloriastrasse 30/32, CH-8006 Zürich, Switzerland; Klinik für Infektionskrankheiten und Spitalhygiene, UniversitätsSpital Zürich, Universität Zürich, Rämistrasse 100, 8091 Zürich, Switzerland; Present Address: Institut für Medizinische Virologie, Universität Zürich, Winterthurerstrasse 190, CH-8057 Zürich, Switzerland; Present Address: Unilabs, Ringstrasse 12, 8600 Dubendorf, Switzerland

**Keywords:** Broad-range fungal ITS PCR, Mycosis, Fungal infection, Surgical site infections, Antifungal

## Abstract

**Background:**

Broad-range fungal inter spacer region (ITS) polymerase chain reaction (PCR) has been evaluated for the detection and identification of fungi in clinical specimens from severely immunocompromised patients, but not in non-selected patients. Thus, the aim of this study was to compare the diagnostic performance of ITS PCR with that of fungal culture and to investigate its clinical impact on the diagnosis of fungal infections in non-immunocompromised patients. The corresponding patients’ data were retrieved by detailed medical chart reviews.

**Results:**

Results from 251 specimens showed a high concordance of 89.6 % for ITS PCR and fungal culture. The analytical sensitivity and specificity of ITS PCR considering culture as gold standard were 87.7 and 90.3 %, respectively, the positive and negative predictive value (PPV and NPV) were 76 and 95.5 %, respectively. Assessing the clinical probability of a fungal infection based on detailed chart reviews, PCR had a clinical sensitivity of 88.9 %, a specificity of 86.3 %, a PPV of 64.0 % and a NPV of 96.6 %. The overall performance of fungal broad-range PCR was similar to that of culture.

**Conclusions:**

Our data show that, in non-selected and non-immunocompromised patients, the performance of ITS PCR is similar to that of culture for detecting fungal infections, not the least because sensitivity of culture in patients under antifungal treatment is surprisingly high. Compared to culture, PCR has the advantage of a rapid time-to-result (approximately two working days), proper identification of rare pathogens, prompt initiation of a species-targeted antifungal treatment, and prospects for automation.

**Electronic supplementary material:**

The online version of this article (doi:10.1186/s12866-016-0752-1) contains supplementary material, which is available to authorized users.

## Background

The epidemiology of fungal infections has seen significant changes over the last decades [1, 2]. In addition, fungal infections are increasing due to more aggressive chemotherapies, immunosuppressive therapeutics, and biologicals interfering with the innate or adaptive immune system [3]. Fungal infections are associated with high morbidity and mortality [4, 5].

The clinic of fungal infections is often subtle and potentially masked by bacterial infection(s) or comorbidities. Clinicians consider and treat invasive fungal infections (IFI) mainly in patients with prolonged and severe neutropenia [6], and in seriously ill patients with extensive candida colonization [7]. In these selected patient groups, decision to treat can be based on criteria established by the European Organisation for Research and Treatment of Cancer (EORTC) or using candida colonization indices (CCI). However, in less selected patient groups, suspicion of fungal infection mainly develops in patients with signs of infection and an unfavorable clinical course, despite treatment with antibiotics. No standardized evaluation criteria or protocols exist for these situations and diagnosis relies on a sophisticated work-up including imaging, biopsies, and serological or microbiological analyses. Surgical site infections (SSI) are the third most frequently reported nosocomial infection in hospitalized patients [8], and an increasing number of SSIs are attributable to fungi [9], in particular *Candida albicans* [8, 9]. Compared to patients with suspicion of IFI, an overtreatment is less acceptable in patients with SSI [10, 11].

The gold standard for diagnosing invasive fungal infections is culture combined with histopathology [12, 13]. Biochemical assays such as 1, 3-beta-D-glucan or galactomannan, which are based on the detection of released fungal products in the blood stream are of value for diagnosing IFIs [14], but have no value for local infections. [15]. DNA based assays excel by their speed and the ability to identify a broad spectrum of fungi, including rarely encountered species [16, 17]. The analytical and clinical sensitivity of ITS PCRs has been previously investigated in selected patient cohorts with a high likelihood of IFI e.g., hemato-oncologic patients [18–23]. Studies determining the diagnostic value of ITS PCR in less selected patient groups are limited. These patients do not fit into the category of severely immunosuppressed patients, but are potentially at risk to suffer from localized fungal infections such as patients with SSI. The goal of this study was to evaluate the analytical and clinical sensitivity and specificity of ITS PCR in patients without overt immunosuppression in comparison to culture, and to explore its value for diagnosis of fungal infections in daily practice.

## Methods

### Study design, clinical specimens and medical record review

We performed a retrospective data analysis of clinical specimens from all patients hospitalized on surgical wards for which fungal culture and ITS PCR was requested, including patients surgically treated by ear/nose/throat specialists (ENTs), ophthalmologists and dermatologists at the University Hospital of Zurich (USZ). The USZ is a tertiary care 850-bed academic center in Switzerland. The specimens were mainly from primarily sterile body sites but also from the eye and from the ear-nose-throat area. All specimens were analyzed by microscopy, conventional culture methods and PCR based on the ITS region. The clinical specimens were collected over a period of three years. The sole exclusion criterion was insufficient chart documentation for the clinical diagnosis of fungal infection. The study was done according to good clinical practice.

Diagnosis of fungal infection was done in the synopsis of disease history, clinical picture and microbiological work up (microscopy, culture, and PCR), and each patient was then categorized into definite fungal infection or no infection. Data were obtained by medical chart review and synopsis was done by a panel of 2 senior infectious disease specialists (S.K.R. and R.F.S.) and 2 microbiologists (A.Z. and G.V.B.) leading to an expert opinion. Data was collected on underlying disease, clinical course of disease and interventions, clinical signs and symptoms of inflammation, laboratory values (leucocytes, CRP, PCT), additional diagnostics (radiology, pathology, and serology) microbiological findings (microscopy, culture, serology), and prior anti-infective treatment. The patients eventually recruited had no overt immunosuppression; in particular no patient was on glucocorticosteroids, drugs interfering with T-cell function or on biologicals.

### Microbiological analyses

#### Phenotypic methods

Clinical specimens were analyzed by microscopy and culture methods for the presence of fungi as described previously [24–26]. Phase contrast light microscopy of clinical samples was performed after potassium hydroxide treatment. Specimens were cultured on general mycology media (Sabouraud dextrose agar containing gentamicin and chloramphenicol, and brain heart infusion (BHI) agar; Becton Dickinson AG, Allschwil, Switzerland) and on fungal selective media (Chromagar, and Mycosel; Becton Dickinson AG) for a maximum of 3 weeks at 25 °C and were regularly examined for growth by eye. Subcultures for identification were done as follows: (i) *Aspergillus* spp. on malt yeast agar [13] at 25, 35, and 42 °C; (ii) mucorales on potato carrot agar at 25, 37, 40, 45, 50, and 56 °C; (iii) all other molds on Sabouraud dextrose agar containing gentamicin and chloramphenicol at 25 and 35 °C and on Mycosel and potato carrot agar, both at 25 °C (temperatures as routinely used in our clinical laboratory). Phenotypic identification was based on macro- and micro-morphological criteria [26].

#### Genotypic methods

DNA extraction from clinical samples (stored at −20 °C) was performed with an EZ1 DNA Tissue Kit (Qiagen, Hombrechtikon, Switzerland) following the manufacturer’s instructions. DNA extracts were eluted in 50 μl of PCR-grade water (Limulus amebocyte lysate [LAL] water; Lonza, Walkersville, MD) of which 5 μl was tested undiluted and in a 1:5 dilution to reduce possible inhibitors of DNA polymerase activity. PCR was performed using a Veriti PCR System (Life Technologies, Zug, Switzerland) in a final volume of 23 μL containing 3 mmol/L MgCl2, 0.5 μmol/L of each primer ITS1 and ITS4 [27], 2 μL of LightCycler FastStart DNA Master SYBR Green I (LightCyler™ reagents; Roche, Rotkreuz, Switzerland) and 5.0 μL of DNA extract. Cycling parameters included an initial heating for 10 min at 37 °C, denaturation for 5 min at 95 °C, 40 cycles of 1 min at 94 °C, 1 min at 48 °C, and 1 min at 72 °C, and final elongation of 10 min at 72 °C. Subsequently, the reaction mixtures were purified with the QIAquick PCR purification kit with a final elution volume of 50 μL (Qiagen, Hombrechtikon, Switzerland). Amplification products were visualized by polyacrylamide gel electrophoresis combined with silver staining. Subsequently, a semi nested PCR was performed using 2.0 μL of the purified reaction mixture and primers ITS3 and ITS4, with the following PCR cycling parameters; 10 min at 37 °C, denaturation for 5 min at 95 °C, 30 cycles of 1 min at 94 °C, 1 min at 50 °C, and 1 min at 72 °C, and final elongation of 10 min at 72 °C. After purification with the QIAquick PCR purification kit, sequencing was performed with primer ITS4 and ITS3 [27] using the BigDye kit (Life Technologies, Zug, Switzerland) and an automated DNA sequencer (ABI Prism 3130-Avant genetic analyzer; Life Technologies, Zug, Switzerland).

Cultured isolates were subjected to DNA extraction using the InstaGene matrix (Bio-Rad, Reinach BL, Switzerland) followed by PCR amplification of the ITS regions and sequencing as described previously [25].

Sequences obtained (covering >90 % of at least one of the ITS regions) were analyzed for homology using GenBank (NCBI) and the SmartGene ITS database (ITS validated database; SmartGene IDNS, Zug, Switzerland) in parallel. Sequence assignment to species and genus level was done according to guidelines published previously (CLSI, 2008) [25].

The inhibition control consisted of amplification of a 422 bp fragment of the *ipaH* gene using *E. coli* K3 chromosomal DNA (~300 bacterial cells per reaction) with primers Shig1 and Shig2 [28]. PCR was performed with a Roche LightCycler 2.0 (Roche Diagnostics; Rotkreuz, Switzerland) following manufacturers guidelines using SYBR green for amplicon detection and 1.0 μL of DNA extract. Reaction conditions were: pre-denaturation at 95 °C for 10 min, 50 cycles of denaturation at 95 °C for 1 s, annealing at 55 °C for 5 s and amplification for 35 s at 72 °C. *Candida albicans* chromosomal DNA was used as a positive PCR control. Buffers, PCR reagents, and elution column solutions were routinely tested for fungal DNA contamination. If fungal identification in a clinical sample was identical to the DNA contamination in one of the controls, the sample was considered PCR negative. In addition, samples were strictly considered negative if 1) the specimen did not show a distinct PCR fragment on polyacrylamide gel electrophoresis and 2) identification pointed to a known environmental contaminant.

### Statistics

We used the 2 × 2 contingency table to calculate sensitivity, specificity, positive and negative predictive values [29]. An overall Friedman (a non-parametric repeated measure ANOVA test) was applied for calculating significance between results of different methods [29].

## Results

### Clinical samples from surgical patients

**Fig. 1:**
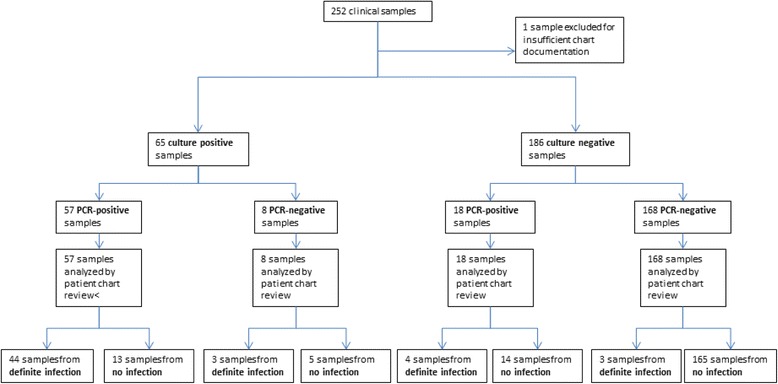
Enrollment of specimens

In total, 251 clinical samples of 163 patients were included in the study (Fig. 1): tissues (*n* = 92), swabs (from wounds, *n* = 66; sternum, *n* = 5; eye, *n* = 27; ear, *n* = 4), and liquid specimens (aspirates, *n* = 36; ascites, *n* = 18; drain fluids, *n* = 3). Specimens were collected at different surgical wards: visceral surgery (*n* = 80), cardiac surgery (*n* = 51), thoracic surgery (*n* = 30), ophthalmology (*n* = 29), traumatic surgery (*n* = 16), plastic surgery (*n* = 16), otorhinolaryngology (*n* = 14), neurosurgery (*n* = 7), dentofacial surgery (*n* = 4), dermatology (*n* = 3), and urology (*n* = 1).

### Analytical sensitivity and specificity of ITS PCR compared with conventional culture

Conventional fungal culture and histopathology are the gold standard for the microbiological diagnosis of fungal infections. Here we evaluated the concordance between ITS PCR and culture. We assigned 49 specimens producing an ITS sequence as negative since these specimens did not show a distinct PCR fragment on polyacrylamide gel electrophoresis and identification pointed to known environmental contaminants (Additional file 1: Table S1); most of these species were *Malassezia* spp. (*n* = 16) and *Cladosporium* sp. (*n* = 12).

We found a concordance of 89.6 % between PCR and culture results (Tables 1 and 2). Doing both, i.e., PCR and culture increased the diagnostic yield from 86 to 92 %. This increase was not significant (overall Friedman test: x^2^ (2, *N* = 36) =2.00, *P* = 0.556). Discordant results were observed in 26 samples with 8 (3.2 %) culture-positive but PCR-negative samples and 18 (7.2 %) culture-negative but PCR-positive samples. The analytical sensitivity of the ITS PCR using culture as the gold standard was 87.7 %, the analytical specificity was 90.3 % (Table 2), the positive predictive value was 76.0 %, and the negative predictive value 95.5 %. The majority of species identified in the specimens by culture and/or PCR were *Candida* spp. (*n* = 54) and *Aspergillus* spp. (*n* = 12) (Table 1). The other species identified were *Saccharomyces cerevisiae* (*n* = 7), *Cladosporium* sp. (*n* = 3), *Rhodotorula minuta* (*n* = 3), *Paecilomyces* sp. (*n* = 2), *Penicillium* sp. (*n* = 3), *Acremonium* sp. (*n* = 1), *Colletotrichum* sp. (*n* = 1), *Mucor* sp. (*n* = 1), *Rhizopus* sp. (*n* = 2), *Tulasnella* sp. (*n* = 1), and *Yarrowia lipolytica* (*n* = 1). Six specimens grew more than one fungal species (Table 1). Microscopy-positive specimens gave mostly (35/37) a culture-positive/PCR-positive result, while this was much lower for microscopy-negative specimens: 22/46 microscopy-negative specimens were culture and PCR-positive, 7/46 were culture-positive/PCR-negative, and 17/46 were culture-negative/PCR-positive (Table 1).

**Table 1:** Fungal species identified by culture and/or broad-range PCR; specimens *n* = 83

	Culture positive, PCR positive	57	Culture positive, PCR negative	8	Culture negative, PCR positive	18
Microscopy positive (*n* = 37)	*A. fumigatus*	3	*A. niger*	1	*A. fumigatus*	1
	*C. albicans*	13				
	*C. albicans* ^a^ *and C. glabrata* ^b^	2				
	*C. albicans* ^a^ *, C. lusitaniae* ^b^ *and S. cerevisiae* ^b^	1				
	*C. albicans* ^a^ *and Penicillium sp.* ^b^	1				
	*C. glabrata*	4				
	*C. norvegensis*	1				
	*C. parapsilosis*	2				
	*C. tropicalis*	1				
	*Colletotrichum sp.*	1				
	*Paecilomyces sp.*	1				
	*Rhizopus sp.*	1				
	*S. cerevisiae*	4				
	Total	35		1		1
Microscopy negative (*n* = 46)	*C. albicans*	11	*A. fumigatus*	1	*Acremonium sp.*	1
	*C. albicans* ^a^ *and C. lusitaniae* ^b^ *and S. cerevisiae*	1	*C. glabrata*	2	*A. fumigatus*	2
	*C. albicans* ^a^ *and Rhizopus sp.* ^b^	1	*Cladosporum sp.* ^c^	3	*A. glaucus*	2
	*C. glabrata*	5	*Penicillium sp.* ^c^	1	*A. nidulans*	1
	*C. kefyr*	1			*Aspergillus sp.*	1
	*C. lusitaniae*	1			*C. albicans*	2
	*Penicillium sp.*	1			*C. lipolytica*	1
	*S. cerevisiae*	1			*Mucor sp.*	1
					*Paecilomyces farinosus*	1
					*Rhodotorula minuta*	3
					*Tulasnella sp.*	1
					*Yarrowia lipolytica*	1
	Total	22		7		17

^a^detected by PCR and culture

^b^detected by culture from a specimen that grew more than one fungal species

^c^these cultures were positive in culture by a single colony, or only in enrichment cultures

**Table 2:** Fungal ITS PCR compared to conventional culture

		Culture	
		+	−
PCR	+	57 (22.7 %)	18 (7.2 %)
	−	8 (3.2 %)	168 (66.9 %)

A total of 251 clinical specimens were included in the study. For fungal identification see Table 1. *Abbreviations*: −, negative; +, positive. Culture was considered as the gold standard

Analytical sensitivity: 87.7 %, Positive predictive value (PPV): 76.0 %

Analytical specificity: 90.3 %, Negative predictive value (NPV): 95.5 %

For discrepant results (*n* = 7), i.e., when species identification by PCR was different from culture, we considered the culture result as true positive and categorized the PCR positive sample in the overall analysis as culture-positive/PCR-negative. However, 4/7 samples, which were negative by PCR, showed only one single fungal colony on the agar plate or were positive only in enrichment cultures (3 *Cladosporium* sp., 1 *Penicillium* sp.) (Table 1). Since microscopy was negative in these specimens, a contamination seemed likely. Considering these 4 samples as culture negative, the analytical sensitivity of the PCR increased to 93.4 %, the specificity 90.5 %, the positive predictive value 76.0 % and the negative predictive value 97.7 %, respectively (Additional file 1: Table S2). Notably, there was only one culture-positive but PCR negative sample, which showed mold hyphae by microscopy. The negative PCR result most likely reflects partial inhibition of PCR (approximately 40 % as determined by RT PCR) caused by traces of the black pigment produced by *A. niger*.

### Clinical sensitivity and specificity of ITS PCR compared with a composite diagnostic measure combining clinic and microbiology data

Here, we examined the performance of broad-spectrum PCR versus a composite diagnostic measure consisting of clinical findings and microbiological results. 54/251 (21.5 %) patient samples from 35 patients categorized as definite fungal infection were identified (Additional file 1: Table S3). 7/54 specimens were culture negative and 47/54 were culture positive. The clinical sensitivity of fungal culture was 87.0 %, the specificity 90.9 %, the positive predictive value (PPV) 72.3 % and the negative predictive value 96.2 % (Table 3). 4/7 culture-negative specimens were PCR-positive with 3/7 *A. fumigatus* and 1/7 *C. albicans*. (Additional file 1: Table S4a). These four culture-negative/PCR-positive specimens were from three different patients. In two of the three patients fungal infection was known (aspergillosis, candidemia) and specimen collection was performed under antifungal therapy. The third patient (with post-operative intracranial abscess after meningioma operation) was not under antifungal therapy at the time of specimen collection; microscopy showed hyphae in one of two samples and ITS PCR identified *A. fumigatus* in both samples. 3/7 cases categorized as definite fungal infections were culture- and PCR-negative. In these patients, microscopy was also negative. All of these patients were under antifungal treatment (Additional file 1: Table S4b).

**Table 3:** Fungal culture compared to composite diagnostic measure for fungal infection including clinical findings and microbiological results (number of samples *n* = 251)

		Fungal infection	
		+	−
Culture	+	47	18
	−	7	179

Clinical sensitivity: 87.0 %, Positive predictive value (PPV): 72.3 %

Clinical specificity: 90.9 %, Negative predictive value (NPV): 96.2 %

*Abbreviations*: −, negative; +, positive

48/54 specimens from definite fungal infection cases were PCR-positive and 6/54 specimens were PCR-negative. In 2/6 PCR-negative cases, culture gave a positive result. The clinical sensitivity of broad-range PCR was 88.9 %, the specificity 86.3 %, the positive predictive value (PPV) 64.0 % and the negative predictive value (NPV) was 96.6 % (Table 4).

**Table 4:** Fungal ITS PCR compared to composite diagnostic measure for fungal infection including clinical findings and microbiological results (number of samples *n* = 251)

		Fungal infection	
		+	−
PCR	+	48	27
	−	6	170

Clinical sensitivity: 88.9 %, Positive predictive value (PPV): 64.0 %

Clinical specificity: 86.3 %, Negative predictive value (NPV): 96.6 %

*Abbreviations*: −, negative; +, positive

36/54 specimens were obtained from patients, who received antifungal treatment for 1 to 48 days (median 10.5 days) prior to sample collection. Notably, fungi were recovered by culture in 31 of those 36 (86 %) specimens (Table 5), 5/36 specimens from patients under antifungal therapy were culture-negative. In 2 of the 5 cases, broad-range PCR was positive. Overall, broad-range PCR was positive in 31/36 specimens from patients under antifungal therapy.

**Table 5:** Fungal ITS PCR and conventional culture results for specimens (*n* = 54) from patients with fungal infection - anti-fungal treatment versus no treatment

	Culture positive	Culture negative
PCR positive	44	4
Under anti-fungal therapy	29	2
No anti-fungal therapy	15	2
PCR negative	3	3
Under anti-fungal therapy	2	3
No anti-fungal therapy	1	0

### Microscopy

All specimens with both culture and PCR negative results were microscopy negative. All specimens positive by microscopy were culture and/or PCR positive (Table 1). For microscopy positive samples ITS PCR provides a rapid mean of species identification (sensitivity of PCR for microscopy positive samples 97.3 %).

## Discussion

The goal of this study was to evaluate the performance of ITS PCR for diagnosis of fungal infections in patients from surgical wards without overt immunosuppression. We found a high concordance between PCR and culture results regarding the analytical sensitivity and specificity as well as the positive and negative predictive value. We used a synopsis of clinical findings and laboratory results to categorize patients into definite fungal infection or no infection. On the basis of these composite diagnostic measures the clinical sensitivity, specificity, PPV, and NPV of ITS PCR and culture were in a similar range (compare Tables 3 and 4). When considering patients under antifungal treatment, we were surprised that PCR did not add much to the sensitivity of culture for diagnosing fungal infections. Indeed, a similar number of specimens were positive by culture and/or PCR. The main advantage of PCR based detection is its diagnostic speed, its prompt identification even of rare species, and its ability to allow rapidly for a species-targeted treatment.

A major disadvantage of ITS PCR concerns the frequent contamination of reagents and materials with traces of fungal DNA [30]. As per the ubiquitous nature of fungi virtually every reagent may show low-level contaminations, e.g., primers, taq polymerase, etc., or contamination becomes introduced during sampling. Strict precautions are necessary, including careful quality control of the reagents, but environmental contamination remains a problem. We used two criteria to recognize environmental contamination and to strictly categorize samples as negative: 1) the specimen did not produce a distinct PCR fragment on polyacrylamide gel electrophoresis, and 2) the species identified pointed to a known environmental contaminant. Using these criteria, we are confident that we have excluded PCR results which were due to contaminations.

The analytical sensitivity of ITS PCR is in the range of 65 % to over 90 % [31, 32] in studies with highly selected patient groups, namely severely immunocompromised patients, most of them with hemato-oncologic diseases and diagnosed with either possible or probable IFI according to the EORTC. However, other patients are also at risk of fungal infections, especially after surgical interventions [9]. Breach in the skin, a hyper-inflammatory state and an excessive use of antibiotics [9] may render those patients susceptible to fungal infections. Diagnosis of fungal infections is particularly delicate under these circumstances and relies on suspicion and, the results of microbiological investigations, namely microscopy and culture [12, 13].

Here we evaluated the value of ITS PCR for patients mainly from surgical wards including patients hospitalized for diseases of the eye, ear/nose/throat or skin. Samples studied included tissue specimens, swabs, and liquid specimens. We found that the analytical sensitivity of the ITS PCR considering culture as the gold standard was 87.7 %, the analytical specificity 90.3 %, respectively. The concordance between these two methods was as high as 89.6 %. Thus we got overall similar results as in previous studies with hemato-oncological or otherwise severely immunocompromised patients [19]. We observed discordant results in either direction, i.e.*,* culture-positive/PCR-negative (3.2 %) and culture-negative/PCR-positive (7.2 %). Because of the high concordance between culture and ITS PCR, performing both only minimally adds to the likelihood to diagnose fungal infections. The diagnosis of fungal infections has to integrate various elements, i.e., clinic data and information as well as results from microbiological investigations. The lack of a gold standard, independent of culture and microscopy, hampers the evaluation of new diagnostic methods. Our patient series included 7 culture-negative specimens from patients, whom we diagnosed to suffer from fungal infection based on history and clinics, a number too low for assessing the diagnostic value of PCR in culture-negative fungal infections.

Notably we observed sustained culture positivity despite anti-fungal treatment. This may be explained by the in general weak potency of antifungal drugs [33–35], empirical treatment not covering the fungal species eventually identified (12/31), specimens obtained for microbiological workup shortly after start of anti-fungal treatment (10/31 within 3 days), or poor drug penetration into surgical site infection. This contrasts to bacterial infections where broad-range bacterial PCR aids significantly in identifying pathogens in patients treated with antibiotics [36].

## Conclusions

In summary, in non-selected patients without overt immunosuppression, ITS PCR has a sensitivity and specificity similar to that of conventional culture, but excels by its speed (standardized time-to-result of 2 working days) and identification of less frequently observed or emerging fungal pathogens, allowing for rapid species-targeted treatment. In addition, and in contrast to culture based detection and identification of fungi, molecular diagnostic procedures can readily be implemented in strategies for full lab automation.

## Abbreviations

BHI, brain heart infusion; CCI, candida colonization indices; CRP, C-reactive protein; ENTs, ear/nose/throat specialists; EORTC, European Organisation for Research and Treatment of Cancer; IFI, invasive fungal infections; ITS, inter spacer region; NPV, negative predictive value; PCR, polymerase chain reaction; PCT, procalcitonin; PPV, positive predictive value; SSI, surgical site infections

## Additional file

Additional file 1: MCRO-D-16-00030 R2 Supplemental data Rampini et al. The supplemental data consist of four tables. **Table S1.** Compilation of false positive PCR results categorized as contaminants; these specimens did not produce a distinct PCR fragment on polyacrylamide gel electrophoresis and identification pointed to known microbiological (environmental) contaminants. **Table S2.** Fungal ITS PCR compared to conventional cultures (*n* = 251). Four microscopy- and PCR-negative but culture positive specimens as included in Table 2, were categorized as culture-negative since cultures were only positive by a single fungal colony or only after enrichment culture; thus, microbiological contamination was highly likely. **Table S3.** Compilation of the patients with fungal infection (*n* = 54), including microbiological data, clinical situation and antifungal therapy. **Table S4.** Overview of culture negative fungal infections subdivided in (1) culture negative, broad-range fungal PCR positive fungal infections and (2) culture negative, broad-range fungal PCR negative fungal infections, including clinical information and antifungal therapy. (DOCX 63 kb)
