# Supplementary material for: Similar efficacy of broad-range ITS PCR and conventional fungal culture for diagnosing fungal infections in non-immunocompromised patients
Source: BMC Microbiol. 2016 Jun 28;16:132. doi: 10.1186/s12866-016-0752-1 (PMC4924236; doi:10.1186/s12866-016-0752-1)
Supplement: Additional file 1: — MCRO-D-16-00030 R2 Supplemental data Rampini et al. The supplemental data consist of four tables. Table S1. Compilation of false positive PCR results categorized as contaminants; these specimens did not produce a distinct PCR fragment on polyacrylamide gel electrophoresis and identification pointed to known microbiological (environmental) contaminants. Table S2. Fungal ITS PCR compared to conventional cultures (n = 251). Four microscopy- and PCR-negative but culture positive specimens as included in Table 2, were categorized as culture-negative since cultures were only positive by a single fungal colony or only after enrichment culture; thus, microbiological contamination was highly likely. Table S3. Compilation of the patients with fungal infection (n = 54), including microbiological data, clinical situation and antifungal therapy. Table S4. Overview of culture negative fungal infections subdivided in (1) culture negative, broad-range fungal PCR positive fungal infections and (2) culture negative, broad-range fungal PCR negative fungal infections, including clinical information and antifungal therapy. (DOCX 63 kb) [file 12866_2016_752_MOESM1_ESM.docx]

Supplemental Data

Table S1. Compilation of false positive PCR results categorized as contaminants; these specimens did not produce a distinct PCR fragment on polyacrylamide gel electrophoresis and identification pointed to known microbiological (environmental) contaminants.

| **Identification by PCR** | **Number of specimens**  **(n=49)** |
| --- | --- |
| *Malassezia restricta* | 12 |
| *Cladosporium* sp. | 12 |
| *Alternaria* sp. | 3 |
| *Candida guilliermondii* | 2 |
| *Epicoccum* sp. | 2 |
| *Malassezia globosa* | 2 |
| *Aspergillus penicilloides* | 1 |
| *Cryptococcus victoriae* | 1 |
| *Dioszegia hungarica* | 1 |
| *Chaetothyriales* sp. | 1 |
| *Galactomyces geotrichum* | 1 |
| *Gymnopus dryophilus* | 1 |
| *Hyphodontia paradoxal* | 1 |
| *Humicola fuscoatra* | 1 |
| *Malassezia* sp. | 1 |
| *Malassezia sympodialis* | 1 |
| *Myrmecridium* sp. | 1 |
| *Ophiostoma* sp. | 1 |
| *Saccharomyces cerevisiae* | 1 |
| *Stereum* sp. | 1 |
| *Truncatella angustata* | 1 |
| *Varicosporium* sp. | 1 |

**Table S2.** Fungal ITS PCR compared to conventional cultures (n=251). Four microscopy- and PCR-negative but culture positive specimens as included in Table 2, were categorized as culture-negative since cultures were only positive by a single fungal colony or only after enrichment culture; thus, microbiological contamination was highly likely.

|  |  | **Culture** | |
| --- | --- | --- | --- |
|  |  | **+** | **−** |
| **PCR** | **+** | 57 (22.7%) | 18 (7.2%) |
|  | **−** | 4 (1.6%) | 172 (68.5%) |

Analytical sensitivity 93.4% Positive predictive value (PPV) 76%

Analytical specificity 90.5% Negative predictive value (NPV) 97.7%

**Table S3.** Compilation of the patients with fungal infection (n=54)

| **Patient number** | **Sample number** | **Clinical specimen** | **Microscopy** | **Culture** | **Broad range PCR** | **Antifungal therapy**  (number of days under antifungal therapy^1^) | **Clinical situation** |
| --- | --- | --- | --- | --- | --- | --- | --- |
| 1 | 1 | Aspirate | Yeasts | *C. glabrata* | *C. glabrata* | FLC (10) | Perforation of Barrett’s esophagus with mediastinitis and pleura empyema |
| 2 | 2 | Sternal wound swab | Yeasts | *C. albicans* | *C. albicans* | No | Open chest treatment after complicated valve reconstruction |
| 3 | 3 | Ascites | Pseudohyphae | *C. albicans* | *C. albicans* | FLC (11) | Open abdomen treatment after small bowel resection with subsequent leakage |
| 4 | 4 | Ear swab | Hyphae | *A. fumigatus* | *A. fumigatus* | No | Chronic otitis media |
| 5 | 5 | Aspirate | Yeasts | *C. albicans* | *C. albicans* | CASP, FLC (6) | Pancreatitis necroticans |
| 6 | 6.1 | Tissue | Yeasts | *C. albicans* | *C. albicans* | CASP (3) | Esophageal perforation with pleura empyema in a patient with Morbus Crohn |
|  | 6.2 | Wound swab | Pseudohyphae | *C. albicans* | *C. albicans* | CASP, FLC (9) |  |
| 7 | 7.1 | Wound swab | Yeasts | *S. cerevisiae* | *S. cerevisiae* | No | Ruptured epiphrenic esophageal diverticulum with pleura empyema |
|  | 7.2 | Tissue | Yeasts | *S. cerevisiae* | *S. cerevisiae* | No |  |
|  | 7.3 | Tissue | Yeasts | *S. cerevisiae* | *S. cerevisiae* | No |  |
| 8 | 8.1 | Aspirate | Yeasts, pseudohyphae | *C. albicans, C. glabrata* | *C. albicans* | FLC (1) | Intraabdominal abscess after hemihepatectomy for liver metastases of a stromal tumor |
|  | 8.2 | Aspirate | Negative | *C. glabrata* | *C. glabrata* | FLC (21) |  |
|  | 8.3 | Aspirate | Negative | *C. glabrata* | *C. glabrata* | FLC (21) |  |
| 9 | 9 | Wound swab | Yeasts | *C. albicans* | *C. albicans* | FLC (2) | Small bowel leakage in patient with ovarial carcinoma |
| 10 | 10.1 | Aspirate | Yeasts | *C. albicans* | *C. albicans* | AFG, FLC (5) | Small bowel perforation after hemi-hepatectomy for hepatocellular carcinoma |
|  | 10.2 | Wound swab | Yeasts, pseudohyphae | *C. albicans* | *C. albicans* | AFG, FLC (13) |  |
|  | 10.3 | Wound swab | Negative | *C. glabrata* | *C. glabrata* | AFG, FLC (48) |  |
|  | 10.4 | Wound swab | Yeasts | *C. glabrata* | *C. glabrata* | AFG, FLC (48) |  |
|  | 10.5 | Wound swab | Yeasts | *C. glabrata* | *C. glabrata* | AFG, FLC (48) |  |
| 11 | 11 | Wound swab | Yeasts, pseudohyphae | *C. albicans* | *C. albicans* | FLC (1) | Ischemic bowel perforation |
| 12 | 12 | Eye swab | Yeasts | *C. parapsilosis* | *C. parapsilosis* | No | Keratitis |
| 13 | 13 | Eye swab | Hyphae | *Paecilomyces* sp. | *Paecilomyces lilcacinus* | VRC (1) | Endophthalmitis after cataract operation |
| 14 | 14 | Ascites | Yeasts | *C. albicans* | *C. albicans* | No | Necrosis of pancreas after combined renal/pancreas transplantation in a patient with diabetes mellitus type 1 |
| 15 | 15 | Sternal wound swab | Yeasts | *C. albicans* | *C. albicans* | No | Sternal infection after mitral valve replacement |
| 16 | 16 | Eye swab | Hyphae | *Lecytophora* sp. | *Lecytophora* sp. | No | Contact-lens related infectious keratitis |
| 17 | 17.1 | Tissue | Hyphae | *A. fumigatus* | *A. fumigatus* | VRC (2) | Decortication of pleura empyema in a patient with broncho-alveolar carcinoma |
|  | 17.2 | Tissue | Hyphae | *A. fumigatus* | *A. fumigatus* | VRC (2) |  |
|  | 17.3 | Tissue | Negative | Negative | Negative | VRC (16) |  |
|  | 17.4 | Tissue | Negative | Negative | *A. fumigatus* | VRC (16) |  |
| 18 | 18.1 | Wound swab | Yeasts, pseudohyphae | *C. albicans*  *C. lusitaniae*  *S. cerevisiae* | *C. albicans,* | No | Duodenal perforation |
|  | 18.2 | Wound swab | Negative | *C. albicans, C. lusitaniae, S. cerevisiae* | *C. albicans* | FLC (5) |  |
| 19 | 19 | Tissue | Yeasts | *C. norvegensis* | *C. norvegensis* | No | Colon perforation |
| 20 | 20.1 | Tissue | Negative | *C. albicans*  *Rhizopus* sp. | *C. albicans* | AMB, CASP, FLC (24) | Duodenal perforation |
|  | 20.2 | Ascites | Hyphae | *Rhizopus* sp. | *Rhizopus microsporus* | AMB, CASP, FLC (24) | Duodenal perforation |
| 21 | 21 | Aspirate | Yeasts | *C. albicans* | *C. albicans* | CASP (1) | Postoperative drainage at day 4 after heart transplantation |
| 22 | 22.1 | Aspirate | Negative | *S. cerevisiae* | *S. cerevisiae* | AFG, CASP, FLC (40) | Postoperative drainage after hemi-hepatectomy for a Klatskin tumor |
|  | 22.2 | Aspirate | Negative | Negative | Negative | AFG, CASP, FLC (48) |  |
|  | 22.3 | Aspirate | Yeasts | *S. cerevisiae* | *S. cerevisiae* | AFG, CASP, FLC (48) |  |
|  | 22.4 | Aspirate | Negative | Negative | Negative | AFG, CASP, FLC (48) |  |
| 23 | 23 | Ear swab | Hyphae | *A. niger* | Negative | No | Cholesteatoma |
| 24 | 24.1 | Tissue | Hyphae | Negative | *A. fumigatus* | No | Debridement of a postoperative abscess after resection of meningioma. |
|  | 24.2 | Tissue | Negative | Negative | *A. fumigatus* | No |  |
| 25 | 25 | Tissue | Negative | *C. albicans* | *C. albicans* | No | Sternal infection after aortic valve replacement |
| 26 | 26 | Aspirate | Negative | *C. kefyr* | *C. kefyr* | AFG, FLC (7) | Retroperitoneal hematoma after rupture of an iliacal aneurysm |
| 27 | 27.1 | Tissue | Negative | *C. albicans* | *C. albicans* | No | Sternal infection after operative correction of atrial septal defect |
|  | 27.2 | Tissue | Negative | *C. albicans* | *C. albicans* | No |  |
| 28 | 28 | Wound swab | Negative | *C. glabrata* | *C. glabrata* | CASP, FLC (37) | Open abdomen treatment in abdominal graft infection |
| 29 | 29 | Tissue | Negative | *C. glabrata* | *C. glabrata* | CASP, FLC (18) | Sternal infection after aortic valve replacement |
| 30 | 30 | Ascites | Negative | *C. albicans* | *C. albicans* | FLC (1) | Intraabdominal bleeding after hemihepatectomy for Klatskin tumor |
| 31 | 31 | Wound swab | Negative | *C. albicans* | *C. albicans* | FLC (4) | Wound infection after laparatomy |
| 32 | 32 | Sternal wound swab | Negative | *C. albicans* | *C. albicans* | No | Sternal infection after mitral and aortic valve replacement |
| 33 | 33 | Ascites | Negative | *C. glabrata* | Negative | VRC (39) | Septic shock with multi-organ failure and small bowel necrosis |
| 34 | 34 | Tissue | Negative | *C. glabrata* | Negative | FLC (8) | Esophageal perforation with pleural empyema |
| 35 | 35 | Tissue | Negative | Negative | *C. albicans* | FLC (3) | Explantation of infected porth-a-cath |

Abbreviations: FLC, fluconazole; AFG, anidulafungin; AMB, amphotericin B; VRC, voriconazole; CASP, caspofungin

^1^ number indicates sum of days under antifungal therapy with different anti-fungals

**Table S4.** Overview of culture negative fungal infections.

1. Culture negative, broad-range fungal PCR positive fungal infections.

| **Clinical specimen^1^** | | **Microscopy** | **Broad range PCR** | **Clinical setting** | **Antifungal therapy**  (days under antifungal therapy) |
| --- | --- | --- | --- | --- | --- |
| 17.4 | Tissue | Negative | *A. fumigatus* | Diagnosis of aspergillosis by decortication of pleura empyema 14 days prior in patient with broncho-alveolar carcinoma | VRC (16) |
| 24.1 | Tissue | Hyphae | *A. fumigatus* | Débridement of postoperative abscess after resection of meningeoma. | No |
| 24.2 | Tissue | Negative | *A. fumigatus* |  | No |
| 35 | Tissue | Negative | *C. albicans* | Explantation of infected porth-a-cath in patient with Candidemia | FLC (3) |

1. Culture negative, broad-range fungal PCR negative fungal infections**.**

| **Clinical specimen** | | **Microscopy** | **Clinical setting** | **Antifungal therapy**  (days under antifungal therapy) |
| --- | --- | --- | --- | --- |
| 17.3 | Tissue | Negative | Decortication of pleura empyema in a patient with broncho-alveolar carcinoma^2^ | VRC (16) |
| 22.2 | Aspirate | Negative | Postoperative drainage after hemi-hepatectomy for a Klatskin tumor^2^ | AFG, CASP, FLC (48) |
| 22.4 | Aspirate | Negative |  | AFG, CASP, FLC (48) |

^1^Clinical specimen number corresponds to sample number of supplementary table 3. Abbreviations: AFG, anidulafungin; AMB, amphotericin; FLC, fluconazole, VRC, voriconazole

^2^Other samples from these patients were positive for fungi by broad range fungal PCR and/or by culture (see suppl. table 3)
